# Use of Donkey Milk in Children with Cow’s Milk Protein Allergy

**DOI:** 10.3390/foods2020151

**Published:** 2013-05-06

**Authors:** Paolo Polidori, Silvia Vincenzetti

**Affiliations:** 1 School of Pharmacy, University of Camerino, Via Circonvallazione 93, 62024 Matelica (MC), Italy; 2 School of Veterinary Medical Sciences, University of Camerino, Via Circonvallazione 93, 62024 Matelica (MC), Italy; E-Mail: silvia.vincenzetti@unicam.it

**Keywords:** donkey milk, cow’s milk allergy, milk protein fractions

## Abstract

Human breast milk is the best nutritional support that insures the right development and influences the immune status of the newborn infant. However, when it is not possible to breast feed, it may be necessary to use commercial infant formulas that mimic, where possible, the levels and types of nutrients present in human milk. Despite this, some formula-fed infant develops allergy and/or atopic disease compared to breast-fed infants. Cow’s milk allergy can be divided into immunoglobulin IgE mediated food allergy and non-IgE-mediated food allergy. Most infants with cow’s milk protein allergy (CMPA) develop symptoms before 1 month of age, often within 1 week after introduction of cow’s milk-based formula. Donkey milk may be considered a good substitute for cow’s milk in feeding children with CMPA since its composition is very similar to human milk. Donkey milk total protein content is low (1.5–1.8 g/100 g), very close to human milk. A thorough analysis of the donkey milk protein profile has been performed in this study; the interest was focused on the milk proteins considered safe for the prevention and treatment of various disorders in humans. The content of lactoferrin, lactoperoxidase and lysozyme, peptides with antimicrobial activity, able to stimulate the development of the neonatal intestine, was determined. Donkey milk is characterized by a low casein content, with values very close to human milk; the total whey protein content in donkey milk ranges between 0.49 and 0.80 g/100 g, very close to human milk (0.68–0.83 g/100 g). Among whey proteins, α-lactalbumin average concentration in donkey milk is 1.8 mg/mL. The results of this study confirmed the possibility of using donkey milk in feeding children with CMPA.

## 1. Introduction

Adverse reactions to food are currently classified into toxic and non-toxic reactions. Non-toxic adverse reactions to milk are primarily caused by either lactose intolerance or milk allergy. Milk intolerance is due to the inherited lack of the specific enzyme, β-galactosidase that is required to hydrolyze lactose. For lactose intolerance, the most common therapeutic approach excludes lactose-containing milk from the diet.

Cow’s milk protein allergy (CMPA) is defined as an immunological reaction to one or more milk proteins [1]. A variety of symptoms can be suggestive for CMPA. CMPA is suspected clinically in 5%–15% of infants [2], while most estimates of prevalence of CMPA vary from only 2% to 5%. Confusion regarding CMPA prevalence is often due to differences in study population, and a lack of defined diagnostic criteria for CMPA. It is very important to define correct diagnostic criteria, in order to avoid the prescription of a wrong diet for infants [3] which can lead to malnutrition [4].

Cow’s milk is a member of the “Big-8” food allergens that include egg, soy, wheat, peanuts, tree nuts, fish and shellfish in terms of prevalence [5]. The prevalence of CMPA varies with age. CMPA is prevalent in early childhood with reported prevalence between 2% and 6% [6,7] and decreases into adulthood to a prevalence of 0.1%–0.5% [8,9]. It has been suggested that infants have milk allergies because milk is usually the first source of foreign antigens that they ingest in large quantities, and the infant intestinal system is insufficiently developed to digest and immunologically react to milk proteins. When milk is eliminated, the inflammation response is controlled. After several years, oral tolerance is developed, and milk can again be tolerated [10]. Most milk allergic children are considered symptom-free by 3 years of age, but several studies have indicated that older children may also have immune reactions to milk. In adults, CMPA is less common than lactose intolerance [11,12], even though it has been reported that approximately 1% of the adult population has milk-specific IgE antibodies. However, studies on CMPA in adults are scarce. CMPA may develop also when breastfed infants (BF) start to receive cow’s milk formula (CMF) and usually occurs within the first weeks after cow’s milk introduction. Manifestations mainly occur at the level of the digestive tract (50%–60%), the skin (50%–60%) and the respiratory tract (20%–30%); they vary from mild-moderate to severe. Immunologically, CMPA can be IgE or non-IgE mediated [13].

For human beings, cow’s milk represents the most common feeding during the infant weaning, but also the first allergen in life. In many countries, cow’s milk is the most important food allergen in babies and children [14]. Adverse reactions to cow’s milk were found in 2% of babies during the first year of life: 30% of cases at the 1stmonth, 60% before the 3rd and 96% within the 12th [15,16]. Symptoms can even appear during the breast-feeding because newborn reacts against a small amount of caseins present in maternal milk [17]. Children followed for the first 3 years of life, 56% of cases had recovered from cow’s milk allergy at 1-year of age, 77% at 2 years and 87% at 3 years of age [18]. However, allergy can persist for all life time. Considering the possible use of alternative milk sources for human in cases of cow’s milk allergy, the use of goat’s milk should be avoided because of the high risk of cross-reactivity, while mare’s and donkey’s milks, used in popular practice for allergic children, are valid alternative protein sources when appropriately evaluated from the hygienic point of view [19]. The discussion on the use of soy-based infant formula is difficult, since scientific societies have different recommendations. There is a broad consensus on the following statements: the incidence of soy allergy in soy formula-fed infants is comparable to that of CMPA in cows’ milk formula-fed babies [20]. Cross reactivity to soy has been reported in 10% to 35% of infants with CMPA, regardless whether they were positive or negative for specific IgE for CMP. In particular, infants with multiple food allergies and eosinophilic enterocolitis also react to soy protein; therefore, different specialist groups have different standpoints on the use of Soy formula for CMPA, but is generally not recommended before the age of 6 months [20].

The donkey (*Equusasinus*) is a member of the horse family, it worked together with humans for centuries, the most common role was for transport. It still remains an important work animal in the poorer regions. Compared with ruminant’s milk, donkey milk has been studied less in the past, but in the last years research interest and capital investment in donkey milk have increased. The protein composition is significantly different from cow’s milk (Table 1): the total content is lower (1.5–1.8 g/100 g) and quite similar to that of human and mare milk; this condition avoids an excessive renal load of solute [21]. The main difference is the proportion of whey proteins: they are 35%–50% of the nitrogen fraction while they represent only 20% in cow’s milk [22]. Comparing donkey’s and mares milk, the casein to whey protein ratio in mares milk is 0.2:1 immediately post-partum, and changes to 1.2:1 during the first week of lactation [23]. 

**Table 1:** Comparison of chemical composition and physical properties of donkey and human milk [24].

	Donkey	Human
pH	7.0–7.2	7.0–7.5
Protein (g/100g)	1.5–1.8	0.9–1.7
Fat (g/100 g)	0.3–1.8	3.5–4.0
Lactose (g/100 g)	5.8–7.4	6.3–7.0
Ash (g/100 g)	0.3–0.5	0.2–0.3
Total solids (g/100 g)	8.8–11.7	11.7–12.9
Caseins (g/100 g)	0.64–1.03	0.32–0.42
Whey proteins (g/100 g)	0.49–0.80	0.68–0.83

## 2. Experimental Section

Milk samples were collected in a farm located in the center of Italy from 20 Martina Franca breed donkeys at middle lactation stage. The study of donkey milk protein profile was approached using different techniques for protein separation: initially they were based on chromatographic techniques followed by sodium dodecyl sulphate polyacrylamide gel electrophoresis SDS-PAGE [25]. Successively, the milk was analyzed through two-dimensional electrophoresis (2-DE) followed by *N*-terminal sequencing, in order to give a more detailed panoramic view of the proteins that are present in donkey milk [26]. Donkey’s milk casein fraction was also characterized by different chromatographic approaches using an Äkta Purifier HPLC system: ion-exchange chromatography (Mono S HR 5/5 column, GE Healthcare, Uppsala, Sweden, 1.0 mL bed volume), and reversed-phase chromatography by a C4 Prosphere column, (Alltech, Waukegan Rd Deerfield, IL, USA). After chromatography, each protein was subjected to SDS-PAGE. The purified caseins were identified by *N*-terminal sequencing [25].

## 3. Results and Discussion

By cation-exchange chromatography, performed at pH 5 and 7, followed by 15% SDS-PAGE (Mini Protean III apparatus, Bio-Rad, Hercules, CA, USA) it was possible to separate nine peaks that were identified as β-caseins (sequence: REKEELNVSS) and α_S1_-caseins (sequence: RPKLPHRQPE), having different molecular weights. Reversed-phase chromatography on HPLC (RP-HPLC) followed by 15% SDS-PAGE and *N*-terminal analysis was performed on the skimmed donkey’s milk giving as a result three main peaks identified as lysozyme (sequence: KVFSKXELA), α-lactalbumin (sequence: KQFTKXELSQVLXSM), and β-lactoglobulin (sequence: TNIPQTMQ), respectively (Table 2). RP-HPLC was also performed on the donkey’s milk casein fraction after their precipitation from skimmed milk at pH 4.6. Five peaks were recovered each of them submitted to 13% SDS-PAGE and *N*-terminal analysis and the results, showed in Table 2, indicated mainly the presence of α_S1_-caseins and β-caseins.

**Table 2:** Donkey’s milk protein fraction identified by reversed phase chromatography in HPLC [25].

Protein	kDa	*N*-Terminal sequence
Lysozyme	14.60	KVFSKXELA
α-lactalbumin	14.12	KQFTKXELSQVLXSM
β-lactoglobulin	22.40	TNIPQTMQ
αs_1_-casein	33.30	RPKLPHQPE
β-casein	37.50	REKEELNVS

This study revealed the presence of β-caseins (sequence: REKEELNVSS) and αs_1_-caseins (sequence: RPKLPHRQPE), which presented marked homology with αs_1_- and β-caseins from mare’s milk [26], while the presence of other types of caseins, such as αs_2_-, γ- and κ- were not determined in donkey milk. This result shows another high similarity between donkey and human milk: in fact, the presence of αs_2_-caseins in human milk has not been demonstrated [27].

**Figure 1:**
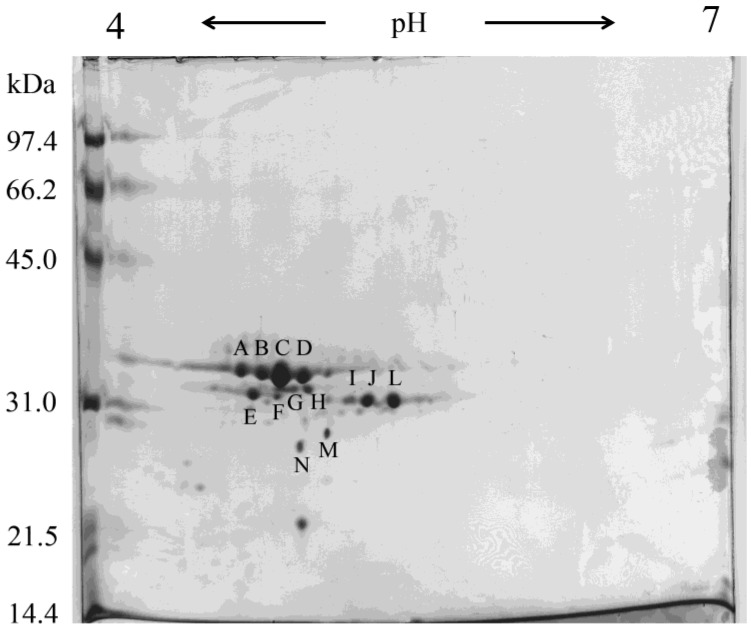
Two-dimensional electrophoresis (2-DE) analysis on donkey milk whole casein.

In order to achieve a better separation of the donkey milk whole casein fractions, we performed a 2-DE analysis in a narrow pH range of 4–7 for the first dimension and in a 13% SDS-PAGE for the second dimension. As a result, about 14 or more major casein spots with molecular mass varying from 27.24 to 33.74 kDa and pI values varying from 4.63 to 5.36 were visualized (Figure 1). The 2-DE map gave a panoramic view on the protein composition of donkey milk: among caseins were found mainly β-caseins (spots A–H) and αs_1_-caseins (spots I–N) and that showed a considerable heterogeneity due to variable degree of phosphorylation and to the presence of genetic variants.

Thanks to RP-HPLC analysis, it was possible also to calculate the lysozyme, β-lactoglobulin and α-lactalbumin concentrations (in mg/mL) at different stages of lactation (60, 90, 120, 160 and 190 days after parturition), the results are shown in Table 3.

**Table 3:** Quantitative determination of lysozyme, β-lactoglobulin, α-lactalbumin in different stages of lactation [25].

Days after parturition	Lysozyme (mg/mL)	β-Lactoglobulin	α-Lactalbumin
60	1.34	Not determined	0.81
90	0.94	4.13	1.97
120	1.03	3.60	1.87
160	0.82	3.69	1.74
190	0.76	3.60	1.63

The amount of lysozyme in donkey’s milk varied considerably during the different stages of lactation, with a mean value of 1.0 mg/mL, and proved to be higher with respect to that in bovine (traces), human (0.12 mg/mL) and goat’s milk (traces), whereas, it was very close to mare’s milk (0.79 mg/mL) [28]. The mean β-lactoglobulin content in donkey’s milk (3.75 mg/mL) was very close to that of bovine milk (3.3 mg/mL) and pony mare’s milk (3.0 mg/mL), whereas in human milk the β-lactoglobulin is absent [27]. The α-lactalbumin content increased in the three months after parturition tothe value of 1.8 mg/mL, close to the α-lactalbumin content in human milk (1.6 mg/mL) but lower thanthe pony mare’s α-lactalbumin content (3.3 mg/mL) [24].

Lactoferrin was purified by a cationic exchange chromatography (Mono S HR5/5 column, GE Healthcare, Uppsala, Sweden) and its identity was confirmed by *N*-terminal sequencing and by western blot analysis using anti-lactoferrin antibodies [29]. The quantitative determination of donkey’s milk lactoferrin gave a result of 0.080 ± 0.0035 g/L, similar to that found in mare (0.1 g/L), cow (0.02–0.2 g/L), goat (0.06–0.40 g/L), and sheep milk (0.135 g/L), but lower when compared with the lactoferrin content in human milk, in which values are usually in the range 1.0–6.0 g/L [25,29].

Lactoperoxidase is a glycoprotein consisting of a single peptide chain with a molecular weight of 78.0 kDa. This enzyme exerts its antimicrobial action through the oxidation of thiocyanate ions (SCN-) by hydrogen peroxide, both present in biological fluids and also in milk. Lactoperoxidase activity in skimmed donkey milk was evaluated by a continuous spectrophotometric rate determination using as substrate 2,2′-azinobis (3-ethylbenzthiazoline-6-sulfonic acid) [30]. In donkey milk, the activity of lactoperoxidase is very low, 4.83 ± 0.35 mU/mL. The enzyme quantification was achieved by a calibration line obtained by plotting the nanograms of peroxidase standard solutions against the enzymatic activity The mean (±SD) concentration of donkey milk lactoperoxidase was calculated to be 0.11 ± 0.027 mg/L, close to the value obtained with human milk (0.77 ± 0.38 mg/L) compared to cow milk [31]. In Table 4 the concentration of three proteins with antimicrobial effect are compared from donkey, human and cow milk. From these data is evinced that human and donkey milk contain considerable amounts of lysozyme and lactoferrin but lactoperoxidase is present only in small amounts.

**Table 4:** Content of lactoperoxidase, lactoferrin and lysozyme from bovine, donkey and human milk [32].

Milk	Lactoperoxidase (mg/L)	Lysozyme (g/L)	Lactoferrin (g/L)
Human	0.77	0.12	0.3–4.2
Donkey	0.11	1.0	0.080
Bovine	30–100	Trace	0.10

## 4. Conclusions

Recent clinical evidence has renewed the interest in donkey milk because of high tolerability in infants with cows’ milk protein allergy. To be successful as a substitute for human milk in infant nutrition, donkey milk must be capable of performing many biological functions associated with human milk. The results obtained in this study can give a valid view about the protein composition of donkey milk. The caseins found were mainly αs_1_- and β-caseins, which showed a considerable heterogeneity. The high lysozyme and α-lactalbumin content found in donkey milk may be responsible for the low bacterial count reported in literature [33]. Lysozyme, lactoperoxidase and lactoferrin have been recognized as antimicrobial and bacteriostatic agents and could be useful to prevent intestine infections in infants. Their action may extend the conservation of fresh donkey milk and the relative potential commercial supply. Donkey milk showed a very high content of lysozyme, while lactoperoxidase was found in a low amount. On the basis of results obtained donkey milk may be considered suitable for feeding young children affected by severe cow’s milk allergy. In the past it has been widely used to replace human milk because its chemical composition and particularly protein content are close to that of human. Great attention must of course be given to the hygienic characteristics of donkey milk production, in order to consider this milk a valid substitute of hydrolyzed proteins or soy-bean derived formulae in the treatment of infants with cow’s milk protein allergy.
